# Impact of Alginate Oligosaccharides on Ovarian Performance and the Gut Microbial Community in Mice with D-Galactose-Induced Premature Ovarian Insufficiency

**DOI:** 10.3390/antiox14080962

**Published:** 2025-08-05

**Authors:** Yan Zhang, Hongda Pan, Dao Xiang, Hexuan Qu, Shuang Liang

**Affiliations:** 1 College of Animal Science and Technology, Jilin Agricultural Science and Technology College, Jilin 132109, China; zhangyan888001@jlnku.edu.cn; 2 Department of Animal Science, College of Animal Sciences, Jilin University, Changchun 130062, China; panhd23@mails.jlu.edu.cn (H.P.); xiangdao22@mails.jlu.edu.cn (D.X.)

**Keywords:** premature ovarian insufficiency, alginate oligosaccharides, oxidative stress, gut microbiota

## Abstract

Premature ovarian insufficiency (POI) is an important factor in female infertility and is often associated with oxidative stress. Alginate oligosaccharides (AOSs), derived from the degradation of alginate, have been demonstrated to have protective effects against various oxidative stress-related diseases. However, the impact of AOSs on POI has not been previously explored. The current study explored the effects of AOSs on ovarian dysfunction in a mouse model of POI induced by D-galactose (D-gal). Female C57BL/6 mice were randomly divided into five groups: the control (CON), POI model (D-gal), and low-, medium-, and high-dose AOS groups (AOS-L, 100 mg/kg/day; AOS-M, 150 mg/kg/day; AOS-H, 200 mg/kg/day). For 42 consecutive days, mice in the D-gal, AOS-L, AOS-M, and AOS-H groups received daily intraperitoneal injections of D-gal (200 mg/kg/day), whereas those in the CON group received equivalent volumes of sterile saline. Following D-gal injection, AOSs were administered via gavage at the specified doses; mice in the CON and D-gal groups received sterile saline instead. AOS treatment markedly improved estrous cycle irregularities, normalized serum hormone levels, reduced granulosa cell apoptosis, and increased follicle counts in POI mice. Moreover, AOSs significantly reduced ovarian oxidative stress and senescence in POI mice, as indicated by lower levels of malondialdehyde (MDA), higher activities of catalase (CAT) and superoxide dismutase (SOD), and decreased protein expression of 4-hydroxynonenal (4-HNE), nitrotyrosine (NTY), 8-hydroxydeoxyguanosine (8-OHdG), and p16 in ovarian tissue. Analysis of the gut microbiota through 16S rRNA gene sequencing and short-chain fatty acid (SCFA) analysis revealed significant differences in gut microbiota composition and SCFA levels (acetic acid and total SCFAs) between control and D-gal-induced POI mice. These differences were largely alleviated by AOS treatment. AOSs changed the gut microbiota by increasing the abundance of *Ligilactobacillus* and decreasing the abundance of *Clostridiales*, *Clostridiaceae*, *Marinifilaceae*, and *Clostridium_T*. Additionally, AOSs mitigated the decline in acetic acid and total SCFA levels observed in POI mice. Notably, the total SCFA level was significantly correlated with the abundance of *Ligilactobacillus*, *Marinifilaceae*, and *Clostridium_T*. In conclusion, AOS intervention effectively mitigates ovarian oxidative stress, restores gut microbiota homeostasis, and regulates the microbiota–SCFA axis, collectively improving D-gal-induced POI. Therefore, AOSs represent a promising therapeutic strategy for POI management.

## 1. Introduction

Infertility is a widespread issue globally, affecting approximately 15% of couples of reproductive age. Premature ovarian insufficiency (POI) is a major factor contributing to female infertility [1]. POI is defined as the cessation of menstruation for more than four months prior to the age of 40, accompanied by elevated follicle-stimulating hormone (FSH) levels reaching menopausal ranges (>40 IU/L) and diminished estradiol (E2) levels (<50 pg/mL) [2]. This condition is associated with a decline in the primordial follicle pool, accelerated follicular atresia, and altered recruitment of dominant follicles [3]. Despite the known risk factors, including genetic, autoimmune, and iatrogenic causes, the exact pathophysiology of POI remains unclear. Current treatments, such as hormone replacement therapy (HRT) and assisted reproductive technology (ART), are often insufficient to fully reverse POI and may pose long-term health risks, such as increased incidences of endometrial and breast cancers [4]. Therefore, it is imperative to investigate additional or supplementary treatment methods to alleviate the complications and adverse effects linked to POI.

D-galactose (D-gal) has been widely used to induce POI in mice. D-gal generates excessive reactive oxygen species (ROS) and results in the accumulation of advanced glycation end products (AGEs) in various tissues, including the ovaries, leading to ovarian aging [5]. This process closely resembles human ovarian aging and has thus become a valuable tool for studying the mechanisms underlying POI. Recent studies have shown that antioxidants, such as resveratrol [6] and curcumin [7], can improve D-gal-induced POI in mice by protecting cells from ROS-induced damage and promoting follicular development and survival. Alginate oligosaccharides (AOSs), low-molecular-weight degradation products of alginate, have garnered attention for their diverse biological activities, including antioxidant [8,9], antitumor [10], anti-inflammatory, and immunomodulatory properties [8]. Previous studies have shown that the continuous intragastric administration of AOSs (100–150 mg/kg/day) for 28 days significantly alleviated D-gal-induced myocardial aging and senile osteoporosis in mice [11,12]. Moreover, AOS administration at 200 mg/kg/day for 14 weeks improved estrogen deficiency-induced sarcopenia by attenuating systemic inflammation [13]. Collectively, these findings suggest that AOS doses ranging from 100 to 200 mg/kg/day are both effective and well tolerated in rodents. Despite these promising characteristics, the potential protective effects of AOSs against D-gal-induced POI remain largely unexplored.

The gut microbiota has emerged as a significant factor influencing the reproductive and endocrine systems. It affects various stages of female reproduction, including serum estrogen levels, fertilization, implantation, embryo migration, and the maturation of ovarian follicles and oocytes [14]. Compared with healthy women, POI patients have been reported to have reduced gut microbial diversity and distinct gut microbiota compositions [15]. Moreover, specific gut microbiota are associated with estrogen levels and the presence of certain metabolic products in the host [16]. These studies highlight the intricate relationship between ovarian function and the gut microbiota, suggesting that modulating the gut microbiota may constitute a novel strategy for POI treatment. However, the potential protective effects of AOSs on POI, particularly their ability to regulate gut microbiota dysbiosis in POI, remain to be elucidated. In this study, we aimed to investigate the protective effects of AOSs on POI using a D-gal-induced POI mouse model. Specifically, we sought to determine the impact of AOSs on ovarian function and oxidative damage-related factors, as well as their effects on the composition of the gut microbiota. By clarifying these aspects, we expect to gain valuable insights into the potential of AOSs as an alternative or complementary therapeutic agent for POI, thereby contributing to a deeper understanding of the pathophysiology underlying this condition.

## 2. Materials and Methods

### 2.1. Animals and Treatment

One hundred healthy female C57BL/6 mice (7–8 weeks old, 16–18 g) were obtained from the Experimental Animal Center of Jilin University, Changchun, China. The mice were maintained under controlled conditions (12 h light/dark cycle, 22 ± 2 °C, 40–70% humidity) and allowed a 7-day acclimatization period. The mice were weighed at baseline and then randomly assigned to five groups (*n* = 20 per group, housed four per cage, with five cages per group): the control (CON), POI (D-gal), low-dose AOS (AOS-L), medium-dose AOS (AOS-M), and high-dose AOS (AOS-H) groups. For 42 consecutive days, mice in the D-gal, AOS-L, AOS-M, and AOS-H groups received daily intraperitoneal injections of D-gal (200 mg/kg/day; G0750, Sigma, St. Louis, MO, USA) to establish the POI model [7,17]. To minimize circadian and order effects, D-gal injections were given between 08:00 and 09:00 each day, and the cage order was rotated daily. Mice in the CON group received equivalent volumes of sterile saline. Following D-gal injection, AOSs were administered by gavage at doses of 100 mg/kg/day (AOS-L), 150 mg/kg/day (AOS-M), and 200 mg/kg/day (AOS-H; Qingdao Hehai Biotechnology Co., Ltd., Qingdao, China). Mice in the CON and D-gal groups received an equal volume of sterile saline by gavage. AOS doses were selected based on previous studies related to inflammation and aging [11,12,13].

### 2.2. Sample Collection

On day 42, a subset of mice from each group was weighed, and fecal samples were collected and frozen at −80 °C for subsequent DNA extraction and analysis of short-chain fatty acids (SCFAs). After anesthesia was induced with pentobarbital sodium (150 mg/kg), blood samples were taken from the mice for hormone analysis. The ovaries were then removed, weighed, and processed: the left ovaries were immersed in 4% paraformaldehyde for histological examination, and the right ovaries were stored at −80 °C for subsequent assays of antioxidant indicators. The remaining mice were subjected to daily vaginal smears for one week to assess the estrous cycle.

### 2.3. Estrous Cycles

To evaluate the estrous cycle stages of the mice, vaginal smears were collected daily for 7 consecutive days, starting 6 weeks after D-gal treatment (*n* = 5 per group). Vaginal lavage samples were subjected to Wright staining (G1040, Beyotime Biotechnology Co., Ltd., Shanghai, China) and observed under a light microscope (Nikon Eclipse, Nikon, Tokyo, Japan).

### 2.4. Hormone and Antioxidant Assays

Mouse serum was obtained from blood samples by centrifugation (900× *g*, 10 min) for hormone assays (*n* = 5 per group). The serum levels of follicle-stimulating hormone (FSH), luteinizing hormone (LH), and estradiol (E2) were measured using the following ELISA kits: FSH (KA2330) and LH (KA2332) from Novus Biologicals (Centennial, CO, USA) and E2 (582251) from Cayman Chemicals (Ann Arbor, MI, USA). For the antioxidant assays, ovarian tissue (*n* = 5 per group) was homogenized in cold Tris-HCl buffer (0.1 M, pH 7.4), centrifuged at 10,000× *g* for 15 min, and then centrifuged at 100,000× *g* for 60 min to eliminate insoluble proteins. The supernatant was collected to measure the activity of catalase (CAT) and superoxide dismutase (SOD) and the content of malondialdehyde (MDA) using SOD (S0101), CAT (S0051), and MDA (S0131S) kits (Beyotime Biotechnology Co., Ltd., Shanghai, China).

### 2.5. Follicle Counting

Ovarian tissues were collected and fixed in 4% paraformaldehyde overnight. After fixation, the tissues were processed for histology according to standard protocols and embedded in paraffin. Five ovarian samples (one per animal) were selected from each group for follicle counting. Each ovary was sectioned at 4 μm and then stained with a hematoxylin and eosin (H&E) kit (G1120, Solarbio, Beijing, China) to reveal the ovarian structures. The classification criteria for follicles were as follows [18]: primordial follicle, characterized by an oocyte enveloped by a single layer of flattened granulosa cells; primary follicle, characterized by an oocyte surrounded by a single layer of cuboidal granulosa cells; secondary follicle, characterized by an oocyte encircled by multiple layers of cuboidal granulosa cells, with no antral cavity present; antral follicle, characterized by an oocyte surrounded by more than four layers of granulosa cells, featuring a prominent antral cavity; and atretic follicle, characterized by a blurred follicular structure, a contracted zona pellucida, and a collapsed follicular wall. In atretic follicles, the oocyte nuclei shrink, the granulosa cell layers diminish in size, the chromosomes and cytoplasm degenerate, and the follicular membrane cells undergo hypertrophy [19].

### 2.6. TUNEL Staining

Apoptotic cells were detected through TUNEL staining of paraffin-embedded sections using a One Step TUNEL Apoptosis Assay Kit (C1089, Beyotime Biotechnology Co., Ltd., Shanghai, China) according to the manufacturer’s guidelines. In detail, TdT labeling buffer was applied to a single section, which was then incubated at 37 °C for 60 min. Following incubation, DAPI was employed as a counterstain on the sections to visualize the nuclei. The nuclei of TUNEL-positive (apoptotic) granulosa cells exhibited green fluorescence, while the DAPI-stained nuclei appeared blue. These samples were observed using an inverted fluorescence microscope (Nikon Eclipse, Nikon, Tokyo, Japan). To achieve consistent and reliable evaluation, four random fields from each slide were examined (with each animal having five slides and each group consisting of five animals, *n* = 5), resulting in a total of 100 random fields (calculated as 5 × 5 × 4 = 100) analyzed per group. The number of TUNEL-positive granulosa cells in the antral follicles per square millimeter (mm^2^) was subsequently quantified using ImageJ software, Version 1.51j8.

### 2.7. Immunohistochemical Staining

Paraffin sections were collected from each mouse ovary (*n* = 5 per group) for immunohistochemical analysis. The sections were deparaffinized in xylene and subjected to antigen retrieval in 10 mM sodium citrate buffer (B0034, Wuhan Boerfu Biotechnology Co., Ltd., Wuhan, China) for 15 min in a microwave oven (M1-L213B, Guangdong Midea, Feshan, China). Once the slides had cooled to room temperature, they were washed three times with PBS (pH 7.4) and subsequently treated with 3% hydrogen peroxide for 25 min in the dark. The sections were then washed again in PBS and blocked with 3% BSA (A8010, Solarbio, Beijing, China) for 30 min. Overnight incubation of the sections was performed with primary antibodies against 8-hydroxyguanosine (1:100, 8-OHdG, sc-393871, Santa Cruz Biotechnology, Dallas, TX, USA), 4-hydroxynonenal (1:400, 4-HNE, BS-6313R, Bioss, Beijing, China), nitrotyrosine (1:200, NTY, sc-71705, Santa Cruz Biotechnology, Dallas, TX, USA), and p16 (1:400, BS-0740R, Bioss, Beijing, China), which were all diluted in PBS. Negative controls were prepared by substituting primary antibodies with PBS alone while keeping all other steps identical. After three washes with PBS, the sections were incubated with the appropriate HRP-conjugated secondary antibody, i.e., goat anti-rabbit IgG (1:2000, ab205718, Abcam, Cambridge, UK) or goat anti-mouse IgG (1:1000, ab6789, Abcam, Cambridge, UK), for 50 min at room temperature. DAB substrate solution (K3468, DAKO, Copenhagen, Denmark) was applied for staining, producing brownish-yellow positive signals. The reaction was terminated with tap water, and the sections were counterstained with hematoxylin for 30 s, differentiated, blued, and rinsed under running water. The sections were observed and photographed under a Nikon Eclipse microscope (Nikon, Tokyo, Japan). The nuclei were stained blue with hematoxylin, while DAB staining resulted in brownish-yellow positive expression. ImageJ software (Version 1.51j8) was used to measure the immunoreactive area (IA) and integrated optical density (IOD) for semiquantification of the staining. The staining intensity (SI) was then calculated as SI = IOD/IA.

### 2.8. Mouse Fecal Microbiota Analysis

Fecal samples from the mice were processed to extract DNA using the DNeasy PowerSoil Kit (47016, Qiagen Enterprise Management Co., Ltd., Shanghai, China) according to the manufacturer’s instructions. The extracted DNA was assessed for quality using 1% agarose gel electrophoresis, and its concentration was quantified with a Nanodrop instrument (NC2000, Thermo Scientific, Waltham, MA, USA). The V3 and V4 regions of the 16S rRNA gene were amplified with the following primers: 341F: 5′-CCTAYGGGRBGCASCAG-3′ and 806R: 5′-GGACTACNNGGGTATCTAAT-3′. The PCR amplification conditions were as follows: initial denaturation at 98 °C for 1 min, followed by 30 cycles of denaturation at 98 °C for 10 s, annealing at 50 °C for 30 s, and extension at 72 °C for 30 s, with a final extension at 72 °C for 5 min. The PCR products were used to construct sequencing libraries using the NEBNext^®^ Ultra™ II DNA Library Prep Kit (E7645L, New England Biolabs, Ipswich, MA, USA) according to the manufacturer’s protocol. The libraries were sequenced on the Illumina MiSeq platform (Illumina, San Diego, CA, USA), generating 250 bp paired-end reads. The sequencing data were analyzed using QIIME2 software, version 2019.4. The sequencing reads were quality-filtered, denoised, and merged using the DADA2 plugin in QIIME2 (version 2019.4) to obtain high-quality amplicon sequence variants (ASVs). Chimeras and singletons were removed with VSEARCH (v2.13.4_linux_x86_64). Taxonomy was assigned against Greengenes2 [20]. Alpha diversity (Chao1, Good_coverage, Simpson, Paith_pd, and Shannon) was calculated on a rarefied ASV table to assess the richness, coverage integrity, evenness, and phylogenetic diversity of the fecal microbiota. Beta diversity was assessed using hierarchical clustering analysis and Bray–Curtis principal coordinate analysis (PCoA), which is based on Bray–Curtis distance matrices. The differences in fecal bacterial communities between various groups were evaluated using Permanova, based on Bray–Curtis distances with 999 permutations, using the ‘vegan’ package in R (version 3.0.3). To pinpoint taxonomic biomarkers associated with POI in mice, we utilized linear discriminant analysis (LDA) effect size (LEfSe) analysis to examine taxa exhibiting substantial abundance disparities between the CON and D-gal groups, employing an LDA score cutoff of greater than 3.5. Taxa with increased abundance in the D-gal group relative to the CON group were designated “D-gal-predominant taxa,” effectively serving as biomarkers for POI-affected mice. Importantly, a parallel comparison was drawn between the AOS and D-gal groups to deduce which genera’s abundance could be influenced by AOS administration. Rarefied tables and summary statistics are provided in the Supplementary Materials.

### 2.9. Short-Chain Fatty Acid (SCFA) Analysis

SCFAs were detected following previously described methods with minor modifications [21,22]. Briefly, for each mouse, approximately 0.2 g of the fecal sample was weighed into a 2 mL centrifuge tube. Distilled water (0.5 mL) was added to each tube and mixed by swirling for 30 s. The samples were then centrifuged at 12,000× *g* for 10 min. A 0.2 mL aliquot of the supernatant was transferred to a new 2 mL centrifuge tube and mixed with 0.1 mL of 15% metaphosphoric acid (7664-38-2, Sinopharm, Beijing, China), 20 μL of a 375 μg/mL 4-methylvaleric acid solution (646-07-1, Sigma, St. Louis, MO, USA) as an internal standard, and 280 μL of ether (60-29-7, Greagent, Shanghai, China). After incubation at 4 °C for 30 min, the mixture was centrifuged at 12,000× *g* for 10 min. Finally, 1 μL of the supernatant was injected into a gas chromatograph (GC) for SCFA analysis. The GC analysis was performed using a GC (Trace 1310, Thermo, Waltham, MA, USA) instrument equipped with an Agilent HP-INNOWAX column (30 m × 0.25 mm ID × 0.25 μm film). The GC conditions were configured such that the initial column temperature of 100 °C was held for 2 min, increased to 200 °C at 15 °C/min, and then maintained for an additional 5 min. The flame ionization detector (FID) was operated at a temperature of 260 °C. The flow rates of hydrogen, air, and nitrogen were set to 35 mL/min, 350 mL/min, and 25 mL/min, respectively. The sample was injected at 260 °C with a split ratio of approximately 25:1, with nitrogen as the carrier gas. Each analysis had a total run time of 12.95 min.

### 2.10. Statistical Analysis

Statistical analyses were conducted using GraphPad Prism (version 8) with one-way ANOVA for group comparisons. When data failed to meet the assumptions of parametric tests, the Kruskal–Wallis test was applied. Statistical significance was set at *p* < 0.05. The data are shown as the means ± SEMs. Normality was evaluated using the Shapiro–Wilk and Kolmogorov–Smirnov tests. Spearman correlation analysis was conducted between the microbiota that showed statistically significant differences between groups and total SCFA levels.

## 3. Results

### 3.1. Determination of the Optimal AOS Concentration for Alleviating D-Gal-Induced POI in Mice

**Figure 1:**
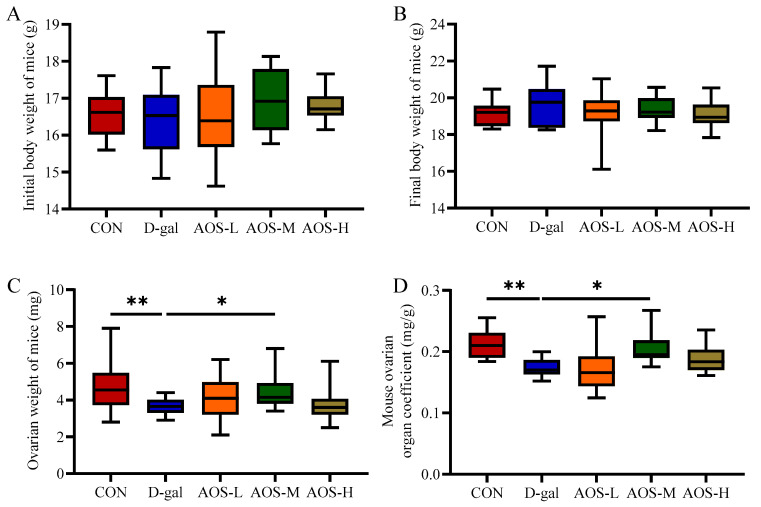
Effects of alginate oligosaccharides (AOSs) on body weight and the ovarian organ coefficient in mice with D-galactose (D-gal)-induced premature ovarian insufficiency (POI). Parameters assessed included the (**A**) initial body weight, (**B**) final body weight, (**C**) ovarian weight, and (**D**) ovarian organ coefficient (*n* = 15–20). The ovarian organ coefficient was calculated using the following formula: ovarian organ coefficient (mg/g) = ovarian weight (mg)/final body weight (g). The data are shown as the means ± SEMs. CON: mice received daily intraperitoneal injections of and gavage with sterile saline for 42 consecutive days; D-gal: mice received daily intraperitoneal injections of D-gal and gavage with sterile saline for 42 consecutive days; AOS-L, AOS-M, and AOS-H: mice received daily intraperitoneal injections of D-gal, followed by gavage with low, medium, and high concentrations of AOSs, respectively, for 42 consecutive days. * *p* < 0.05; ** *p* < 0.01.

To determine the optimal concentration of AOSs for alleviating D-gal-induced POI in mice, various doses of AOSs were administered to POI model mice via D-gal injection. The body weight, ovarian weight, and ovarian organ coefficient were measured to assess the therapeutic efficacy of different AOS doses (Figure 1A–D). The initial and final body weights did not significantly differ among the groups (*p* > 0.05). Compared with the CON group, the D-gal group presented significantly lower ovarian weights and ovarian organ coefficients (*p* < 0.01, *p* < 0.01). Notably, these reductions were significantly mitigated by treatment with a medium dose of AOSs (AOS-M, 150 mg/kg/day, *p* < 0.05). Therefore, based on these findings, the medium AOS dose was determined to be the most effective concentration for alleviating POI in this mouse model.

### 3.2. AOSs Attenuate Ovarian Dysfunction in Mice with D-Gal-Induced POI

**Figure 2:**
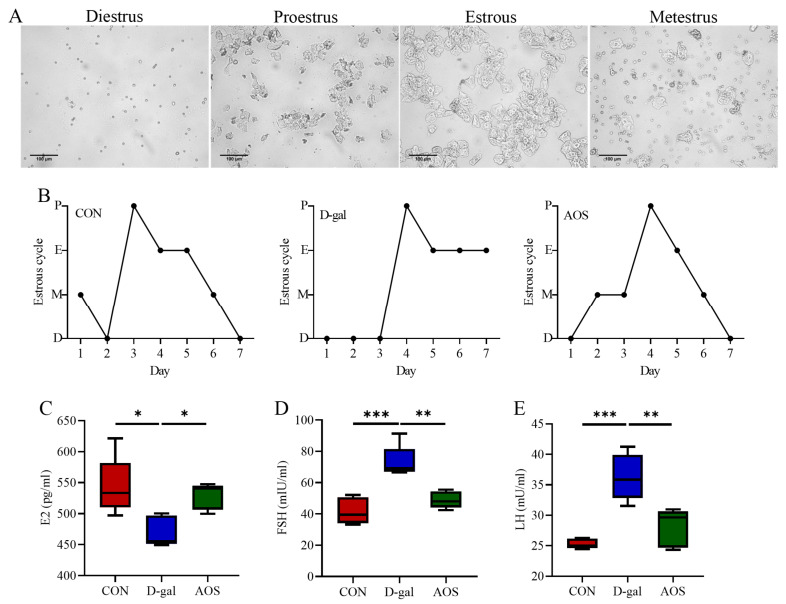
Effects of alginate oligosaccharides (AOSs) on the estrous cycle and hormone levels in mice with D-galactose (D-gal)-induced premature ovarian insufficiency (POI). (**A**) Representative vaginal smears (scale bar: 100 μm). (**B**) Four stages of the estrous cycle (P, E, M, and D indicate proestrus, estrous, metestrus, and diestrus, respectively; *n* = 5). Serum levels of (**C**–**E**) follicle-stimulating hormone (FSH), luteinizing hormone (LH), and estradiol (E2) (*n* = 5). CON: mice received daily intraperitoneal injections of and gavage with sterile saline for 42 consecutive days; D-gal: mice received daily intraperitoneal injections of D-gal and gavage with sterile saline for 42 consecutive days; AOS: mice received daily intraperitoneal injections of D-gal, followed by gavage with medium concentrations of AOSs, for 42 consecutive days. The data are shown as the means ± SEMs. * *p* < 0.05; ** *p* < 0.01; *** *p* < 0.001.

**Figure 3:**
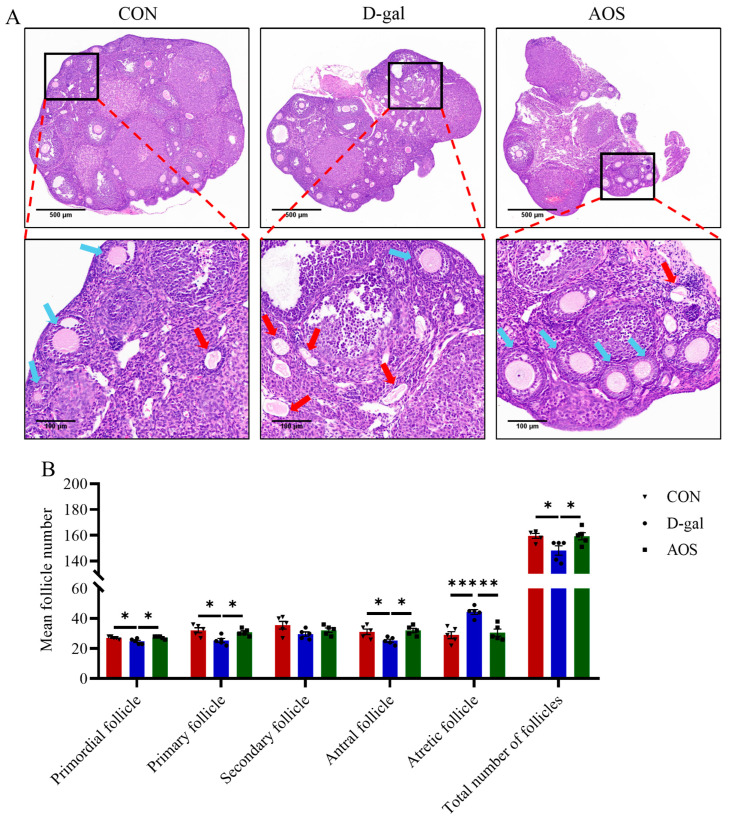
Effects of alginate oligosaccharides (AOSs) on follicular development in mice with D-galactose (D-gal)-induced premature ovarian insufficiency (POI). (**A**) Histological sections of ovaries stained with H&E; red arrows indicate atretic follicles, whereas blue arrows denote other types of follicles; scale bars are 500 μm at 5× magnification and 100 μm at 20× magnification. (**B**) The numbers of follicles at different developmental stages in the ovaries (*n* = 5). The data are shown as the means ± SEMs. CON: mice received daily intraperitoneal injections of and gavage with sterile saline for 42 consecutive days; D-gal: mice received daily intraperitoneal injections of D-gal and gavage with sterile saline for 42 consecutive days; AOS: mice received daily intraperitoneal injections of D-gal, followed by gavage with medium concentrations of AOSs, for 42 consecutive days. * *p* < 0.05; ** *p* < 0.01; *** *p* < 0.001.

The mice in the CON group displayed regular estrous cycles with a duration of 4–5 days, whereas the majority of the mice in the D-gal group were primarily in the diestrus phase. This irregularity in the estrous cycle was significantly ameliorated by AOS treatment (Figure 2A,B). The serum concentrations of critical sex steroid hormones, namely, E2, LH, and FSH, were subsequently quantified (Figure 2C–E). Compared with the CON group, the D-gal group presented markedly elevated levels of serum LH (*p* < 0.001) and FSH (*p* < 0.001) but lower E2 levels (*p* < 0.05). AOS administration significantly attenuated the elevated LH (*p* < 0.01) and FSH levels (*p* < 0.01) while concurrently increasing the serum E2 levels (*p* < 0.05) in POI mice. Moreover, follicle counting was conducted via H&E staining (Figure 3A,B). In the CON group, ovaries contained follicles at various stages of development. In contrast, the D-gal group presented an increased number of atretic follicles (*p* < 0.001), a relative scarcity of primordial (*p* < 0.05), primary (*p* < 0.05), and antral follicles (*p* < 0.05), and a reduced total number of follicles (*p* < 0.05) compared with the CON group. AOS treatment notably enhanced follicular development, bringing the results closer to those observed in the CON group. These findings suggest that the POI model was effectively established and that AOS administration effectively alleviated POI-related symptoms.

### 3.3. AOSs Ameliorate Ovarian Oxidative Stress and Ovarian Cell Apoptosis in Mice with D-Gal-Induced POI

**Figure 4:**
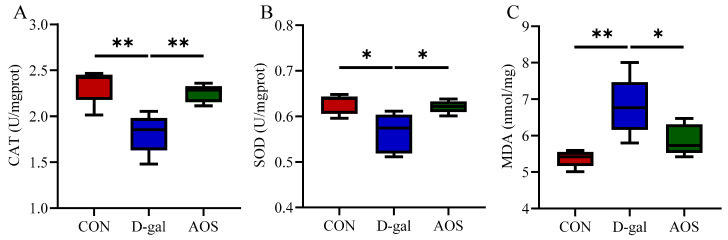
Effect of alginate oligosaccharides (AOSs) on ovarian oxidative stress in mice with D-galactose (D-gal)-induced premature ovarian insufficiency (POI). (**A**–**C**) The activities of catalase (CAT) and superoxide dismutase (SOD), as well as the malondialdehyde (MDA) content, were measured using commercial ELISA kits (*n* = 5). The data are shown as the means ± SEMs. CON: mice received daily intraperitoneal injections of and gavage with sterile saline for 42 consecutive days; D-gal: mice received daily intraperitoneal injections of D-gal and gavage with sterile saline for 42 consecutive days; AOS: mice received daily intraperitoneal injections of D-gal, followed by gavage with medium concentrations of AOSs, for 42 consecutive days. * *p* < 0.05; ** *p* < 0.01.

**Figure 5:**
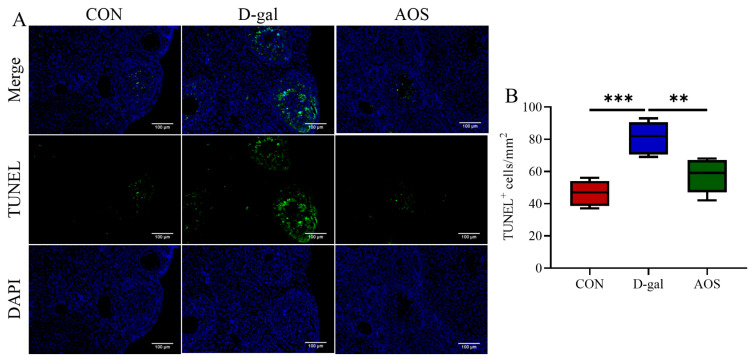
Effect of alginate oligosaccharides (AOSs) on ovarian granulosa cell apoptosis in mice with D-galactose (D-gal)-induced premature ovarian insufficiency (POI). (**A**) Apoptosis was assessed using in situ TUNEL fluorescence, and TUNEL-positive (apoptotic) granulosa cell nuclei were visualized with green fluorescence, whereas nuclei stained with DAPI appeared blue; scale bars: 100 μm at 20× magnification. (**B**) The density of TUNEL-positive granulosa cells within the antral follicles, expressed as the count per square millimeter (mm^2^), was assessed and compared among the three groups (*n* = 5). The data are shown as the means ± SEMs. CON: mice received daily intraperitoneal injections of and gavage with sterile saline for 42 consecutive days; D-gal: mice received daily intraperitoneal injections of D-gal and gavage with sterile saline for 42 consecutive days; AOS: mice received daily intraperitoneal injections of D-gal, followed by gavage with medium concentrations of AOSs, for 42 consecutive days. ** *p* < 0.01; *** *p* < 0.001.

We further evaluated the levels of oxidative stress biomarkers in the ovaries. Compared with the CON group, the D-gal group presented significantly decreased activities of CAT (*p* < 0.01) and SOD (*p* < 0.05), along with a significant increase in the MDA level (*p* < 0.01). In contrast, the AOS group presented significantly greater activities of CAT (*p* < 0.01) and SOD (*p* < 0.05) and lower MDA levels (*p* < 0.05) than the D-gal group (Figure 4A–C). These results highlight the strong antioxidant potential of AOSs. Additionally, we assessed apoptosis in granulosa cells using TUNEL staining, wherein apoptotic granulosa cells were stained green (Figure 5A). The number of apoptotic cells was significantly greater in POI mice than in CON mice (*p* < 0.001). However, AOS treatment significantly reduced the number of apoptotic cells (*p* < 0.01, Figure 5B).

### 3.4. AOSs Ameliorate Ovarian Oxidative Damage and the Expression of Proteins Associated with Ovarian Senescence in Mice with D-Gal-Induced POI

**Figure 6:**
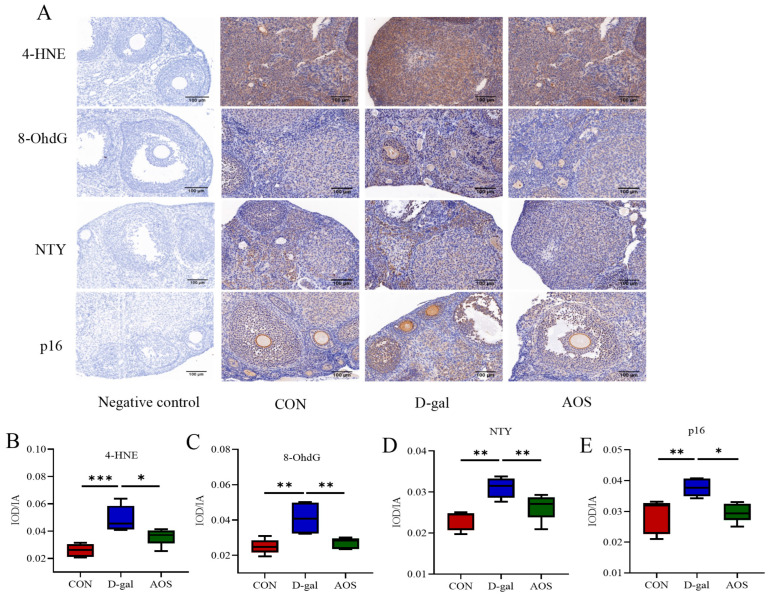
Effects of alginate oligosaccharides (AOSs) on oxidative damage and ovarian senescence-associated protein expression in mice with D-galactose (D-gal)-induced premature ovarian insufficiency (POI). (**A**) The cellular locations of 4-hydroxynonenal (4-HNE), nitrotyrosine (NTY), 8-hydroxydeoxyguanosine (8-OHdG), and p16 were visualized using immunohistochemistry; scale bars: 100 μm at 20 × magnification. The expression levels of 4-HNE (**B**), NTY (**C**), 8-OHdG (**D**), and p16 (**E**) were quantitatively analyzed (*n* = 5). The data are shown as the means ± SEMs. CON: mice received daily intraperitoneal injections of and gavage with sterile saline for 42 consecutive days; D-gal: mice received daily intraperitoneal injections of D-gal and gavage with sterile saline for 42 consecutive days; AOS: mice received daily intraperitoneal injections of D-gal, followed by gavage with medium concentrations of AOSs, for 42 consecutive days. * *p* < 0.05; ** *p* < 0.01; *** *p* < 0.001.

Immunohistochemical analyses were performed to investigate the expression levels of oxidative stress markers (4-HNE, NTY, and 8-OHdG) and the senescence-associated protein p16 in ovarian tissue (Figure 6). Compared with the CON group, the D-gal group presented significantly elevated expression levels of the oxidative stress markers 4-HNE, 8-OHdG, and NTY (*p* < 0.001, *p* < 0.01, *p* < 0.01), indicating increased oxidative stress. Similarly, D-gal treatment also induced a significant increase in the protein expression of the cellular senescence marker p16 (*p* < 0.01). AOS treatment significantly mitigated the oxidative stress and senescence induced by D-gal. Specifically, the expression levels of 4-HNE, 8-OHdG, and NTY were significantly lower in the AOS group than in the D-gal group (*p* < 0.05, *p* < 0.01, *p* < 0.01). Moreover, AOS treatment effectively reversed the D-gal-induced increase in p16 protein expression (*p* < 0.05). These results demonstrate that D-gal induces significant oxidative stress and cellular senescence in ovarian tissue, whereas AOSs exert potent antioxidant and antisenescence effects, thereby alleviating ovarian aging in the mouse model.

### 3.5. AOSs Attenuate Gut Microbiota Dysbiosis in Mice with D-Gal-Induced POI

**Figure 7:**
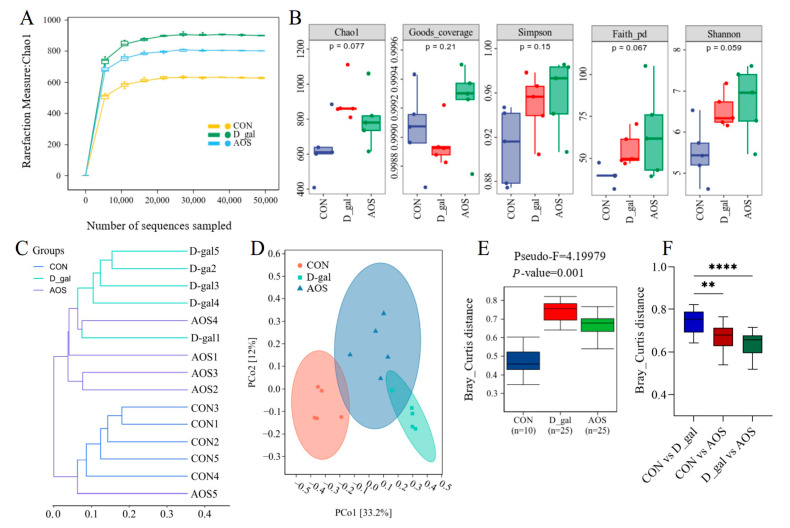
Effects of alginate oligosaccharides (AOSs) on gut microbiota diversity in mice with D-galactose (D-gal)-induced premature ovarian insufficiency (POI). (**A**) Rarefaction curves. (**B**) Alpha diversity was assessed using the Chao1, Good_coverage, Simpson, Paith_pd, and Shannon indices. (**C**,**D**) Beta diversity was measured based on the Bray–Curtis distance and visualized by hierarchical clustering analysis and principal coordinate analysis (PCoA) (*n* = 5). (**E**,**F**) Microbial compositional differences were quantified using the Bray–Curtis distance. CON: mice received daily intraperitoneal injections of and gavage with sterile saline for 42 consecutive days; D-gal: mice received daily intraperitoneal injections of D-gal and gavage with sterile saline for 42 consecutive days; AOS: mice received daily intraperitoneal injections of D-gal, followed by gavage with medium concentrations of AOSs, for 42 consecutive days. ** *p* < 0.01; **** *p* < 0.0001.

The V3-V4 region of the 16S rRNA gene was sequenced in 15 fecal samples. Chao1 curves revealed that sequencing saturation was achieved for samples from the CON, D-gal, and AOS groups (Figure 7A), ensuring comprehensive coverage of bacterial diversity and the identification of nearly all present taxa. The alpha diversity of the gut microbiota was assessed using abundance-based metrics, including the Chao1, Good_coverage, Simpson, Paith_pd, and Shannon indices. The results revealed no significant differences in the alpha diversity indices across the different groups (Figure 7B). These findings indicate that neither D-gal treatment nor AOS intervention had a significant effect on the alpha diversity of the gut microbiota.

We subsequently evaluated the beta diversity of the gut microbiota across different groups of mice using clustering methods and PCoA based on Bray–Curtis distance matrices. The clustering results revealed that samples from the CON and D-gal groups were distinctly separated into two independent clusters, indicating a significant difference in the gut microbiota profile between D-gal and CON mice. Although the AOS group did not fully separate from the D-gal group, it exhibited a certain degree of dissimilarity, with the gut microbiota composition of D-gal mice being more similar to that of CON mice (Figure 7C). These findings suggest that AOS treatment can alter the gut microbiota profile of D-gal mice. Further PCoA and Permanova analysis revealed distinct differences in the gut microbiota composition among the three groups of mice (Figure 7D–F), and AOS supplementation was able to partially restore the compositional differences in the fecal microbiota between D-gal and CON mice.

**Figure 8:**
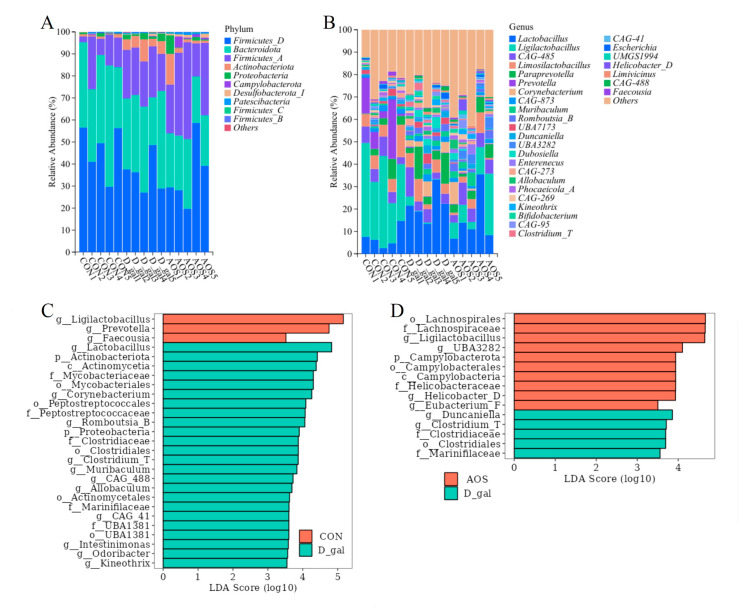
Effects of alginate oligosaccharides (AOSs) on the gut microbiota composition in mice with D-galactose (D-gal)-induced premature ovarian insufficiency (POI). (**A**) Relative abundance of phyla in fecal microbial taxa. (**B**) Relative abundance of genera in fecal microbial taxa. (**C**) Linear discriminant analysis (LDA) effect size (LEfSe) analysis (LDA score > 3.5) highlighting enriched fecal microbial taxa between the CON and D-gal groups. (**D**) LEfSe analysis (LDA score > 3.5) highlighting enriched fecal microbial taxa between the D-gal and AOS groups (*n* = 5). Red bars indicate enrichment in the CON or AOS group, whereas green bars indicate enrichment in the D-gal group. CON: mice received daily intraperitoneal injections of and gavage with sterile saline for 42 consecutive days; D-gal: mice received daily intraperitoneal injections of D-gal and gavage with sterile saline for 42 consecutive days; AOS: mice received daily intraperitoneal injections of D-gal, followed by gavage with medium concentrations of AOSs, for 42 consecutive days.

Taxonomic composition analysis of the fecal microbiota (Figure 8A,B) revealed that the predominant phyla across all mouse fecal samples were *Firmicutes_D*, *Bacteroidetes*, and *Firmicutes_A*. Additionally, the most common genera observed in all the mouse groups included *Lactobacillus*, *Ligilactobacillus*, *CGA485*, *Limosilactobacillus*, *Paraprevotella*, and *Prevotella*. LEfSe analysis (Figure 8C,D) indicated that the relative abundance of four D-gal-predominant taxa (biomarkers of mice with POI), which were assigned to the order *Clostridiales*, the families *Clostridiaceae* and *Marinifilaceae*, and the genus *Clostridium_T*, was significantly reduced in the AOS group. Notably, the genus *Ligilactobacillus*, which was enriched in the CON group, was also enriched in the AOS group. These findings suggest that AOS supplementation may alter the abundance of specific microbiota, potentially mitigating the microbial imbalances associated with POI. This finding suggests that AOSs could help restore a more balanced microbial community, which is essential for overall health.

### 3.6. AOSs Alleviate the Reduction in Fecal SCFA Levels Observed in Mice with D-Gal-Induced POI

**Figure 9:**
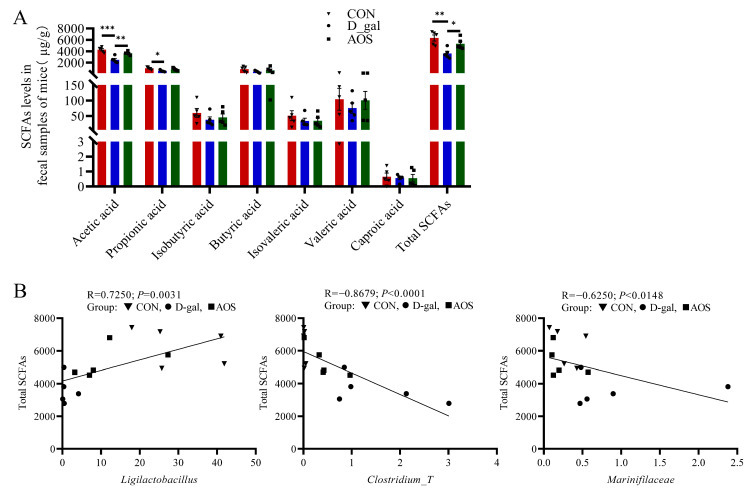
Associations between gut microbial species and total short-chain fatty acid (SCFA) levels. (**A**) SCFA production in fecal samples (*n* = 5). The data are shown as the means ± SEMs. * *p* < 0.05; ** *p* < 0.01; *** *p* < 0.001. (**B**) Scatter plot indicating the Spearman correlation coefficient (with statistical significance at *p* < 0.05) between the total SCFA level and the abundance of *Ligilactobacillus*, *Clostridium_T*, and *Marinifilaceae* across all three groups. CON: mice received daily intraperitoneal injections of and gavage with sterile saline for 42 consecutive days; D-gal: mice received daily intraperitoneal injections of D-gal and gavage with sterile saline for 42 consecutive days; AOS: mice received daily intraperitoneal injections of D-gal, followed by gavage with medium concentrations of AOSs, for 42 consecutive days.

Compared with the CON group, the D-gal group presented markedly lower levels of acetic acid and total SCFAs (*p* < 0.001, *p* < 0.01), and these differences were effectively reversed by AOS treatment (*p* < 0.01, *p* < 0.05). In parallel, the D-gal group presented significantly lower propionic acid levels than did the CON group (*p* < 0.05). Butyric acid mirrored the trend observed for acetic acid, albeit without achieving statistical significance (Figure 9A).

### 3.7. Associations Between the Gut Microbiota and Total SCFA Level

The relationships between the total SCFA level and the abundance of *Ligilactobacillus, Clostridium_T*, and *Marinifilaceae* were examined using Spearman correlation analysis, and the results are depicted in Figure 9B. These analyses indicated that higher levels of *Marinifilaceae* and *Clostridium_T*, along with lower levels of *Ligilactobacillus*, were linked to decreased total SCFA levels in POI mice. After AOS intervention, which altered the abundance of these microbial taxa, the total SCFA level was significantly increased in POI mice.

## 4. Discussion

This study successfully established a mouse model of POI using D-gal, which induced significant decreases in the ovarian organ coefficient, serum E2 level, and number of primordial and primary follicles, which is consistent with previous research [5,7]. D-gal treatment effectively simulates aging-related physiological changes and enhances our understanding of ovarian aging driven by oxidative stress. Given the antioxidant properties of AOSs, we investigated their potential to protect against ovarian aging. In this study, three doses of AOSs were administered orally to POI model mice, with the medium dose yielding the most significant protective effects. We observed that AOS treatment effectively ameliorated estrous cycle disorders; decreased serum LH and FSH levels; increased serum E2 levels; reduced granulosa cell apoptosis; and increased the counts of primordial, primary, antral, and total follicles in D-gal-treated mice. These findings suggest that AOSs can significantly improve ovarian dysfunction in POI mice. Our findings further support those of earlier studies that reported the benefits of antioxidant supplementation in individuals with POI [7,23]. In addition, previous research has demonstrated that AOSs can scavenge oxygen free radicals and mitigate oxidative stress in target organs, thereby conferring a protective effect [8,9]. Here, we found that AOSs significantly reduced ovarian oxidative stress and senescence, as indicated by decreased MDA levels; increased CAT and SOD activities; and downregulated protein expression of 4-HNE, NTY, 8-OHdG, and p16 in ovarian tissue. Therefore, the ability of AOSs to reduce oxidative stress in the ovaries appears to be the key mechanism underlying their protective effects against D-gal-induced POI.

Dysbiosis of the gut microbiota is observed in individuals with POI [15,24], and this correlation is also evident in POI animal models [25]. Our findings revealed significant differences in the fecal microbiota of the mice between the D-gal and CON groups, indicating a disordered gut microbiota in the POI model. Importantly, AOS treatment seemed to restore the fecal microbiota in the D-gal group, with its profile being more similar to that observed in the CON group. Previous studies have shown that gut microbiota β-glucuronidase (gmGUS) can activate estrogen by converting its inactive form to the active form, thereby influencing the host estrogen level [26,27]. Dysbiosis of the gut microbiota has been associated with reduced gmGUS activity and disrupted estrogen metabolism. Considering the essential role of estrogen in ovarian aging, the activity of the gut microbiota is critically important for reproductive health. In this study, we observed significant differences in fecal microbial beta diversity and serum E2 levels between the CON and D-gal groups. However, AOS treatment partially alleviated these differences, further highlighting the complex interplay between ovarian aging and the gut microbiota. However, notably, this study could not completely exclude all potential confounding factors, such as microbial colonization and dietary variability, which may affect the gut microbiota independently of AOS treatment. Although the housing conditions of the experimental mice were strictly controlled, baseline differences in the gut microbiota among individuals may still have been present, and such differences may have influenced the regulatory effects of AOSs on the gut microbiota. Future studies could mitigate this impact by increasing the sample size or using mice from the same litter. Furthermore, during the experimental period, all the mice were fed the same standard feed to reduce the influence of the diet on the gut microbiota, but the composition of the feed may indirectly affect the structure of the microbiota. Subsequent studies can further explore the interaction between different feed components and AOSs, as well as the specific effects of this interaction on the gut microbiota and ovarian function.

Moreover, alterations in the abundance of specific gut microbiota taxa have been correlated with the clinical manifestations of POI [28]. Our results revealed that the relative abundance of *Clostridiales*, *Clostridiaceae*, *Marinifilaceae*, and *Clostridium_T* was notably greater in the D-gal group. In contrast, *Ligilactobacillus*, which was prevalent in the CON group, was also enriched in the AOS group. *Ligilactobacillus*, a genus with a rich taxonomic profile, holds promise for diverse applications. Research has shown that *Ligilactobacillus* strains possess antioxidant capabilities, allowing them to scavenge reactive oxygen species and counteract oxidative stress-induced cellular damage [29]. This protective mechanism could be particularly beneficial for ovarian cells, including oocytes and granulosa cells, helping to maintain optimal ovarian function. Furthermore, specific strains of *Ligilactobacillus salivarius* have demonstrated the ability to degrade and bind estrogen in vitro, thereby modulating estrogen levels and potentially influencing ovarian function [30]. This estrogen-regulating activity may offer relief from reproductive conditions linked to estrogen imbalances. *Ligilactobacillus* also contributes to gut health by producing SCFAs through the fermentation of dietary fiber [31]. SCFAs play a vital role in maintaining gastrointestinal well-being and modulating immune responses [32,33]. A decrease in SCFA levels can have systemic health effects, which may indirectly impact ovarian function. This aligns with findings from a mouse model of POI, where significant changes in the gut microbiota composition and reduced SCFA levels were observed [25]. Overall, these findings indicate that *Ligilactobacillus* may exert beneficial effects on ovarian function and female reproductive health, with antioxidant and estrogen-modulating properties that could safeguard ovarian cells, optimize the reproductive milieu, and enhance fertility outcomes. The family *Clostridiaceae* is nested within the order *Clostridiales*, and the genus *Clostridium_T* is a member of this family. These taxonomic units are home to a diverse array of bacteria with extensive metabolic capabilities. Many of these bacteria are beneficial and contribute to gut health by breaking down a variety of organic substances, including carbohydrates, proteins, and fats [34]. However, these groups also harbor several pathogenic strains. For example, certain species of the genus *Clostridium* can overgrow under specific conditions, leading to microbial imbalances and triggering a range of diseases [35]. Notably, *Clostridium perfringens* produces toxins that can cause severe conditions such as necrotizing enteritis and gas gangrene [36]. Additionally, *Clostridium difficile* is known for its antibiotic resistance and association with intestinal inflammation, posing a significant public health challenge [37]. These examples highlight the potential harmful functions of certain *Clostridium* strains, primarily through their pathogenicity and toxin production capabilities. In our study, we observed a significant increase in the abundance of *Clostridiales*, *Clostridiaceae*, and *Clostridium_T* in the gut of mice with D-gal-induced POI. Moreover, *Clostridium_T* was significantly negatively correlated with the total SCFA level. Similarly, previous studies have reported that in certain disease states, there is a negative correlation between the abundance of the genus *Clostridium* and SCFA levels [38]. This relationship may be mediated by the inhibitory effects of SCFAs on the growth of *Clostridium* and the indirect impact of *Clostridium* on SCFA production, highlighting its significant clinical relevance to gut health and disease [39]. Although no direct studies have reported a connection between *Clostridium_T* and POI, the interplay between the microbiota and host health supports the hypothesis that such a relationship may exist. Specifically, bacteria of the *Clostridium_T* genus may increase oxidative stress levels in the host by producing toxins, triggering inflammatory responses, or inhibiting SCFA generation. These mechanisms can damage ovarian cells, particularly oocytes and granulosa cells, leading to ovarian dysfunction. Additionally, certain strains of *Clostridium_T* may disrupt the host immune system, causing immune dysregulation that increases the risk of autoimmune oophoritis and contributes to POI. *Marinifilaceae*, a family within the phylum *Bacteroidetes*, is essential for degrading and transforming particulate organic matter in natural environments. However, its enrichment in the human gut is linked to gut microbiota dysbiosis, which can affect ovarian health via the microbiota–gut–ovary axis. For example, one study showed that in a chicken model of oxidative stress induced by tert-butyl hydroperoxide treatment, the relative abundance of *Marinifilaceae* in the gut significantly increased, which was correlated with reduced SCFA levels and altered gut microbial diversity [40]. These findings may explain the changes in the gut microbiota composition observed in the D-gal group in our study, characterized by elevated *Marinifilaceae* abundance and reduced SCFA levels. Overall, certain microbial taxa, such as *Ligilactobacillus*, *Clostridium_T*, and *Marinifilaceae*, have been shown to be critical in influencing ovarian health and the development of POI. These findings emphasize the need to address the potentially detrimental effects of these microbes, particularly *Clostridium_T* and *Marinifilaceae*, to effectively prevent and manage POI. Additionally, probiotics such as *Ligilactobacillus*, which can regulate the gut microbiota and SCFA levels, may offer protective benefits against POI.

## 5. Conclusions

AOSs obviously improved ovarian function in mice with D-gal-induced POI, possibly through decreasing ovarian oxidative stress and rebalancing the gut microbiota. However, the underlying mechanisms still need to be fully clarified. Future studies could investigate whether AOSs directly act on ovarian cells by reducing apoptosis and restoring hormone production through in vitro experiments, such as exposing granulosa cells to oxidative stress conditions and treating granulosa cells with AOSs. In addition, the role of the microbiota in the recovery of ovarian function can be confirmed through methods such as fecal microbiota transplantation, antibiotic clearance, or sterile animal models, and the potential role of the gut microbiota in the biosynthesis of SCFAs and estrogen metabolism can be further explored. Such strategies could provide a more nuanced understanding of how oxidative stress, the microbiome, and ovarian physiology intersect. Taken together, our findings provide a preliminary basis for considering AOSs as a potential treatment for POI, but their effectiveness and optimal dosing in humans require thorough clinical evaluation.

## Data Availability

Data generated/analyzed during this study are available from the corresponding author upon reasonable request. Raw amplicon sequence data and associated metadata are deposited in the NCBI SRA database (accession number PRJNA1290631).

## Supplementary Materials

The following supporting information can be downloaded at: https://www.mdpi.com/article/10.3390/antiox14080962/s1, Table S1: Sequence data statistics of fecal samples of mice.
