# Supplementary material for: Impact of Alginate Oligosaccharides on Ovarian Performance and the Gut Microbial Community in Mice with D-Galactose-Induced Premature Ovarian Insufficiency
Source: Antioxidants (Basel). 2025 Aug 5;14(8):962. doi: 10.3390/antiox14080962 (PMC12382774; doi:10.3390/antiox14080962)
Supplement: Supplementary file 1 [file antioxidants-14-00962-s001.zip › Supplementary Materials.pdf]

Supplementary Materials

Impact of Alginate Oligosaccharides on Ovarian Performance and the Gut Microbial Community in Mice with D-galactose-Induced Premature Ovarian Insufficiency

Yan Zhang [1], Hongda Pan [2], Dao Xiang [2], Hexuan Qu [2,*] and Shuang Liang [2,*]

1 College of Animal Science and Technology, Jilin Agricultural Science and Technology College, Jilin, 132109, China

2 Department of Animals Sciences, College of Animal Sciences, Jilin University, Changchun, 130062, China

* Correspondence: quhx21@mails.jlu.edu.cn (Hexuan Qu); liangshuang85@jlu.edu.cn (Shuang Liang)

Table S1 Sequence data statistics of fecal samples of mice.

	CON (n=5)	D-gal (n=5)	AOS (n=5)
No. of input paired reads	82,943 ±2,083	80,473 ± 797.6	74,918 ± 2,127
Quality-filtered reads	76,383 ± 1,821	74,004 ± 826.7	69,223 ± 1,953
Denoised reads	75,197± 1,731	72,265 ± 777.4	67,859 ± 1,808
Merged reads	70,843 ± 1,479	65,842 ± 927.9	63,183 ± 1,327
Chimera-filtered sequences	62,770 ± 1,327	59,200 ± 1,789	59,372 ± 1,546
Singleton-filtered sequences	62,699 ± 1,321	56,111 ± 1.807	5,9330 ± 1,550

The data are shown as the means ± SEMs. CON: mice received daily intraperitoneal injections of and gavage with sterile saline for 42 consecutive days; D-gal: mice received daily intraperitoneal injections of D-gal and gavage with sterile saline for 42 consecutive days; AOS: mice received daily intraperitoneal injections of D-gal, followed by gavage with medium concentrations of AOSs, for 42 consecutive days.
